# A forced titration study of the antioxidant and immunomodulatory effects of Ambrotose AO supplement

**DOI:** 10.1186/1472-6882-10-16

**Published:** 2010-04-30

**Authors:** Stephen P Myers, Lesley Stevenson, Phillip A Cheras, Joan O'Connor, Lyndon Brooks, Margaret Rolfe, Paul Conellan, Carol Morris

**Affiliations:** 1 Australian Centre for Complementary Medicine Education and Research, A Joint Venture of the University of Queensland and Southern Cross University, Lismore, Australia; 2 NatMed-Research Unit, Research Cluster for Health and Wellbeing, Southern Cross University, Lismore, Australia; 3 Graduate Research College, Southern Cross University, Lismore, Australia; 4 Centre for Phytochemistry and Pharmacology, Southern Cross University, Lismore, Australia; 5 The New Zealand Institute for Plant and Food Research Limited, Auckland, New Zealand

## Abstract

**Background:**

Oxidative stress plays a role in acute and chronic inflammatory disease and antioxidant supplementation has demonstrated beneficial effects in the treatment of these conditions. This study was designed to determine the optimal dose of an antioxidant supplement in healthy volunteers to inform a Phase 3 clinical trial.

**Methods:**

The study was designed as a combined Phase 1 and 2 open label, forced titration dose response study in healthy volunteers (n = 21) to determine both acute safety and efficacy. Participants received a dietary supplement in a forced titration over five weeks commencing with a no treatment baseline through 1, 2, 4 and 8 capsules. The primary outcome measurement was *ex vivo *changes in serum oxygen radical absorbance capacity (ORAC). The secondary outcome measures were undertaken as an exploratory investigation of immune function.

**Results:**

A significant increase in antioxidant activity (serum ORAC) was observed between baseline (no capsules) and the highest dose of 8 capsules per day (p = 0.040) representing a change of 36.6%. A quadratic function for dose levels was fitted in order to estimate a dose response curve for estimating the optimal dose. The quadratic component of the curve was significant (p = 0.047), with predicted serum ORAC scores increasing from the zero dose to a maximum at a predicted dose of 4.7 capsules per day and decreasing for higher doses. Among the secondary outcome measures, a significant dose effect was observed on phagocytosis of granulocytes, and a significant increase was also observed on Cox 2 expression.

**Conclusion:**

This study suggests that Ambrotose AO^® ^capsules appear to be safe and most effective at a dosage of 4 capsules/day. It is important that this study is not over interpreted; it aimed to find an optimal dose to assess the dietary supplement using a more rigorous clinical trial design. The study achieved this aim and demonstrated that the dietary supplement has the potential to increase antioxidant activity. The most significant limitation of this study was that it was open label Phase 1/Phase 2 trial and is subject to potential bias that is reduced with the use of randomization and blinding. To confirm the benefits of this dietary supplement these effects now need to be demonstrated in a Phase 3 randomised controlled trial (RCT).

**Trial Registration:**

Australian and New Zealand Clinical Trials Register: ACTRN12605000258651

## Background

There is now substantial evidence that reactive oxygen species (ROS), or oxygen free radicals, are involved in a range of inflammatory diseases [1-3]. Normally ROS are effectively detoxified in the body by the presence of scavenging substances known as antioxidants and antioxidant defence enzymes. Oxidative stress occurs when there is an imbalance between ROS production and the body's intrinsic scavenging capacity leading to an excess of ROS [3]. Individuals who are critically ill are generally exposed to an increase in oxidative stress [4] which has been demonstrated to be proportional to the severity of their condition [5]. One of the reasons that oxidative stress occurs in critical illness, is that the plasma concentrations of antioxidant micronutrients are low. This occurs as a result of fluid losses, low nutrient intakes, dilution of nutrient concentration by resuscitation fluids, and the redistribution of nutrient from plasma to tissues as part of the inflammatory process [6,7].

The low plasma concentration of antioxidant nutrients has led to the postulation that antioxidant therapy may play a role in the treatment of numerous diseases, ranging from acute and chronic inflammation to shock and ischemia/reperfusion injury [1,2,8,9]. Recent research using antioxidant therapies has demonstrated positive effects in both acute[10] and chronic inflammatory conditions [11]. A recent review of the role of oxidative stress-related organ dysfunction in inflammatory and septic conditions demonstrated that only three antioxidant nutrients, selenium, glutamine and eicosapentaenoic acid, have demonstrated clinical benefits and reached the highest level of evidence [7]. The reviewers concluded that other antioxidants are still awaiting well-designed clinical trials.

A range of foods have been found to contain antioxidant constituents which have been demonstrated to effect human antioxidant status. These include spinach [12], strawberries [12], honey [13], soy foods [14] and a variety of edible nuts including pistachios [15], almonds [16] and hazelnuts [17]. Phenolic and polyphenolic compounds are responsible for most of the antioxidant capacity found in fruits, vegetables and most botanical antioxidant supplements [18]. These include quercetin a dietary flavonoid (a polyphenolic) abundant in onions [19] and present in a wide variety of fruits and vegetables and the flavonoid epcatechin found in cocoa, tea and grapes [20].

This present study tested a dietary supplement containing a mixture of antioxidant compounds (Vitamin E and quercetin) and concentrated plant extracts (including green tea and grape extracts) using an open label forced titration design to determine both its acute safety and its antioxidant and immunomodulatory effects in a population of healthy smokers and non-smokers. This is a combined Phase 1 and 2 study aimed at determining the optimal dose to assess in a Phase 3 randomized controlled trial.

## Methods

### Research Design

The trial was an open label forced titration dose response study conducted over 5 weeks in healthy smokers and healthy non-smokers in Lismore, a regional city, in northern New South Wales and was conducted in 2005. In a forced titration study, the study preparation at different doses is given to all subjects. In this study the study preparation was given in increasing doses every week over 5 weeks. In order to test the preparation in healthy volunteers with a low and high exposure to oxidative stress, non-smokers and smokers were selected respectively.

The study was approved by two ethical review panels, the Human Research Ethics Committee of Southern Cross University (Ethics approval number: ECN-04-156) and the University of Queensland Human Research Ethics Committee (Ethics approval number: 200400036). The research was conducted in compliance with Good Clinical Practices (GCP) and in accordance with the guidelines of the Australian National Health and Medicinal Research Council and the Declaration of Helsinki (as revised in 2004). The trial was registered with the Australian and New Zealand Clinical Trials Register (ACTRN12605000258651).

### Participants

A convenience sample healthy individuals, half smokers and half non-smokers, aged between 18 and 50 years were recruited by email from staff and students at Southern Cross University, and from Lismore, and surrounding areas through newspaper advertising, regional radio and television. Individuals were accepted to the study on a first come basis provided they met the study inclusion and exclusion criteria. All participants received a study information sheet outlining the study and signed a consent form agreeing to participate.

A sample size estimate (alpha 0.05 and beta 0.8) based on pilot data from an open label study conducted by Mannatech Inc (Coppell, Texas, USA) in healthy non-smokers required 10 participants to demonstrate changes in the antioxidant status (the primary outcome). In order to assess the effectiveness of the preparation in low and high oxidative stress it was decided to recruit 10 non-smokers and 10 smokers.

Participants were included if they were healthy, and had neither an acute or chronic medical condition. This was determined by a comprehensive general health questionnaire and assessment by the clinical trial nurse (JO), with any concerns resolved by a medical practitioner (SPM). Baseline bloods were also reviewed to ensure subjects with undiagnosed abnormalities were not included. Participants were excluded if they were: taking antioxidant medications and/or other dietary supplements; if they had poor venous access; if they had a history of any auto-immune disorders or diabetes; if they were taking immune suppressant drugs, cytokines, interferon, Echinacea or other immune stimulating herbs; if they had clinically abnormal liver function tests at baseline; were unwilling to have blood taken 6 times during the study; or were unwilling to comply with the study protocols.

### Outcome Measurements

The primary outcome measurement was *ex vivo *changes in serum oxygen radical absorbance capacity. The secondary outcome measures were *in vivo *changes in lymphocyte subsets [Mature T Cells (CD3+), B Cells (CD19+), Helper T cells (CD3+CD4+), Cytotoxic T Cells (CD3+CD8+), Natural Killer (NK) Cells (CD3-CD16+and/orCD56+)], phagocytosis of granulocytes and monocytes, natural killer cell cytotoxicity and cyclooxygenase-2 (COX-2) expression. These immune indices were chosen to explore other biological activities that may be plausibly altered by components within the preparation under investigation.

Two baseline efficacy measurements were taken one week apart prior to the commencement of study medication and subsequent measurements were taken weekly for 4 weeks. Six measurements were taken in total and participants attended 6 clinics during which weight, blood pressure and concomitant medication use was recorded and fasting blood samples collected.

Safety was assessed by actively monitoring adverse events and by a full blood count, liver function tests and determination of urea, creatinine and electrolytes taken at the first baseline measurement and at the conclusion of the study to assess toxicity to the hemopoietic, hepatic and renal system. Vital signs (pulse and blood pressure) and weight were also monitored at every measurement point.

Subjects returned all remaining capsules at each clinic and these were counted as a measure of compliance. The investigator maintained an inventory record of all capsules received and dispensed. It was assumed that capsules not returned were taken.

### Study Supplement and Dosing Schedule

The study product Ambrotose AO^® ^capsules was provided by Mannatech, Incorporated, (Coppell, Texas). The preparation is sold widely in the United States as a dietary supplement. Each capsule contained 18 mg vitamin E as mixed tocopherols (as d-alpha, d-beta, d-delta and d-gamma tocopherols); 113 mg of an antioxidant blend (quercetin dihydrate; grape skin extract; green tea extract; *Terminalia ferdinandiana *[Australian bush plum powder], 331 mg of a proprietary blend of plant polysaccharide and fruits and vegetables powders (aloe vera inner leaf gel, gum acacia, xanthan gum, gum tragacanth, gum ghatti, broccoli, brussel sprouts, cabbage, carrot, cauliflower, garlic, kale, onion, tomato, turnip, papaya and pineapple.

The dosing schedule was one capsule daily for one week after completion of the second baseline measurement. At the end of first week of supplementation, participants then commenced on two capsules daily for the second week, followed by 4 capsules daily for the third week and eight capsules daily for the fourth week. Measurements were taken before and after each increase in dosage.

### Laboratory Assays

#### Serum Oxygen Radical Absorbance Capacity (ORAC)

The ORAC assay employed in this study measured the antioxidant scavenging capacity of serum samples, against peroxyl radicals induced by 2, 2'-azobis (2-amidinopropane) dihydrochloride (AAPH) (Wako, Richmond, VA, USA) at 37°C. Fluorescein sodium salt (C_20_H_12_O_5_·2Na) (Aldrich, St. Louis, MO, USA) was used as the fluorescent probe. The method is based on that of Prior et al. [21] for measuring the antioxidant capacity of plasma and other biological samples.

#### Sample Preparation

Serum samples were stored at -80°C until analysis. Samples were thawed to room temperature, mixed by vortex, and then centrifuged (3 min. at 13 000 *g*). Serum (100 μL) was added to a 1.5 mL micro centrifuge tube with 300 μL Milli-Q water and 400 μL 0.5 M perchloric acid (Sigma, St. Louis, MO, USA), giving a 1 in 8 dilution. Samples were mixed by vortexing, centrifuged (5 min. at 13 000 *g*), and the supernatant was serially diluted 1 in 2 with 75 mM phosphate buffer, pH7.4 (Sigma, St. Louis, MO, USA), giving a dilution series of 1 in 8, 1 in 16, 1 in 32, and 1 in 64. Each dilution was assayed in triplicate.

#### Method

Trolox ((±)-6-Hydroxy-2,5,7,8-tetra-methylchromane-2-carboxylic acid) (Fluka, Buchs, Switzerland), a water soluble analogue of vitamin E, was used as a reference standard. A working Trolox solution (10 μL stock solution, 990 μL perchloric acid precipitating solution) was prepared from a Trolox stock solution (0.01 M, in 75 mM phosphate buffer, pH 7.4). The working solution was then serially diluted 1 in 2 with phosphate buffer (75 mM, pH7.4). A standard curve was established from Trolox standards prepared at 100, 50, 25, and 12.5 μM.

Grape juice (commercially available from supermarket) was included as a control, and was diluted similarly, firstly with perchloric acid precipitating solution, and then serially diluted with phosphate buffer (75 mM, pH 7.4), giving final concentrations of 20, 10, 5, and 2.5 μL/mL.

Briefly, 10 μL fluorescein (6.0 × 10^-7^M), 20 μL samples/standards/control/blank (perchloric precipitating solution) and 170 μL AAPH (20 mM) were added per well. Immediately after loading, the 96 well black fluorescence plate (Greiner, Frickenhausen, Germany) was transferred to the Wallac Victor2 plate reader, preset to heat to 37°C, and the fluorescence was measured every minute for 35 minutes. The fluorescence readings were referenced to solvent blank wells. The final serum ORAC values were calculated using linear regression between the Trolox concentration and the net area under the fluorescein decay curve, and were expressed as micromole Trolox equivalents (TE) per litre of serum.

#### Lymphocyte Subsets

Flow cytometric analysis was used for monitoring the expression of CD3+, CD4+, CD8+, CD19+, CD16+ & CD56+ antigens on peripheral blood lymphocytes (PBL). Staining of PBL was performed by the BD Lyse/No Wash method using MultiTEST IMK kit reagents (BD Biosciences, San Jose, CA, USA).

Briefly, 50 μl whole blood (EDTA) was added to 20 μl of both monoclonal antibodies (CD3/CD8/CD45/CD4 & CD3/CD16+56/CD45/CD19). Tubes were vortexed and incubated for 15 minutes in the dark at room temperature. 450 μl 1× MultiTEST Lysing Solution (BD Biosciences, San Jose, CA, USA). was added to each tube, then tubes were vortexed and incubated for 15 minutes in the dark at room temperature. The samples were then analysed on a BD FACSCalibur flow cytometer, with BD MultiSET software, using excitation wavelengths of 488 nm & 635 nm.

Absolute white blood cell (WBC) and lymphocyte percentage values, obtained from a Beckman Coulter AcT Diff analyser, were used for lymphocyte subset calculations.

#### Phagocytic Activity

Blood samples were assayed for both granulocyte & monocyte phagocytic activity (post E. coli stimulation) by flow cytometry using the Phagotest kit (Orpegen Pharma, Heidelberg, Germany).

E. coli, commercially labelled with fluorescein isothiocyanate (FITC) (Orpegen Pharma, Heidelberg, Germany), was added to aliquots of blood from each lithium heparinised blood sample and incubated for 10 minutes at 37°C. Baseline controls (0°C) were also run for each sample. For each aliquot, the percentage of phagocytes (granulocytes & monocytes) that had ingested the FITC labelled bacteria (E. coli) was then determined by flow cytometry using a BD FACSCalibur instrument.

#### NK Cell Cytotoxic Activity

Peripheral blood mononuclear cells (PBMC) were prepared from each lithium heparinised blood sample using Ficoll-Paque (Amersham Biosciences, Piscataway, New Jersey, USA). K562 cells, an erythroblastic leukaemia cell line (ATCC, Manassas, VA, USA) susceptible to NK cell cytotoxic activity, were pre-labelled with a green fluorescent dye, DiO (Molecular Probes, Carlsbad, CA, USA). The PBMCs for each sample were incubated for two hours (37°C) with the K562 target cells at a ratio of 25:1 (PBMC:K562). After incubation, a cell viability dye, propidium iodide (Molecular Probes, Carlsbad, CA, USA), was added to label the K562 target cells permeabilised by NK activity. A target cell control was also run to monitor spontaneous K562 death. The percentage of dead target cells for each sample was determined by flow cytometry using a BD FACSCalibur instrument. Specific NK cell cytotoxicity was determined as the difference between the percent dead K562 for each test sample and the target cell control. This method is based on that outlined in the NKTest Protocol (Orpegen Pharma).

#### Cox-2 Expression

Blood samples were assayed for monocyte Cox-2 expression post stimulation with E. coli lipopolysaccharide (LPS) (Sigma, St. Louis, MO, USA).

LPS (final concentration 0.01 μg/mL) was added to aliquots of blood from each lithium heparinised blood sample and incubated for two hours at 37°C. Baseline controls (no LPS) were also run for each sample. Samples from each aliquot were then stained with fluorochrome labelled monoclonal antibodies (mAbs) CD14-PerCP (BD Biosciences, San Jose, CA, USA) then intracellularly with Cox-2-PE (BD Biosciences, San Jose, CA, USA). The percentage of monocytes expressing Cox-2 was then determined by flow cytometry using a Becton Dickinson FACSCalibur instrument. This method is based on that of Ruitenberg and Waters [22].

## Results

### Study Participants

The study screened 30 individuals by phone resulting in 24 clinic appointments. Subsequently 22 individuals were enrolled in the study. One participant (47 year old male) did not show after week 3 and was lost to follow up. One subject (43 year old female) was withdrawn at week 5 with diarrhoeal complaint, and one subject (37 year old female) was withdrawn at week 6 with an upper respiratory tract infection. Twenty one (n = 21) data sets were analysed, comprising 9 non-smokers (3 males, 6 females) and 13 smokers (5 males, 8 females). The mean age (± SD) of the participants was 41 years (± 8.55) with a median age of 43.5 years (range 21 - 50 years). The mean age (± SD) of the non-smokers was 42 years (± 7.75) with a median age of 44 years (range 27 - 50 years). The mean age (± SD) of the smokers was 39.5 years (± 9.58) with a median age of 43 years (range 21 - 50 yrs).

### Antioxidant Activity

**Figure 1:**
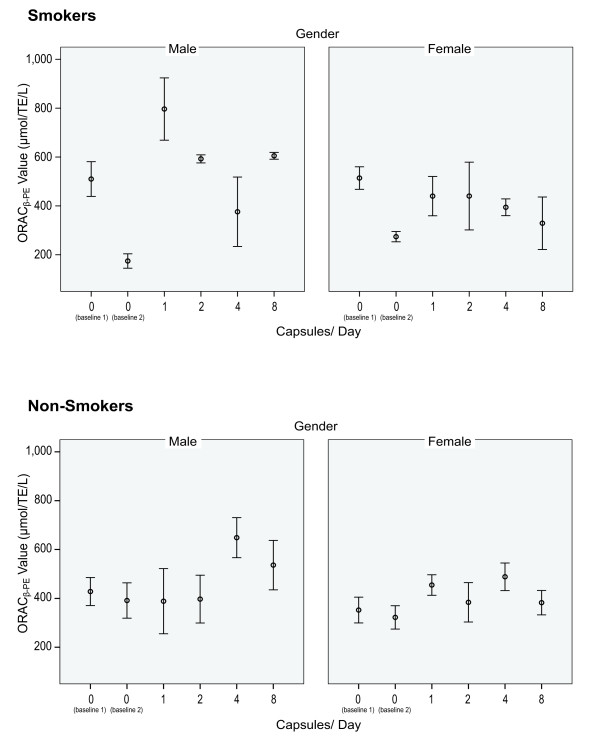
**Serum ORAC - Plots of Means (± SE) by gender and smoking status for each dose**. Baseline 1 and 2 were taken prior to the administration of study preparation.

Descriptive statistics including sample size, mean, standard deviation, minima and maxima for the primary outcome measures of Oxygen Radical Absorption Capacity (serum ORAC) are reported in Table 1 by dosage level, gender and smoking status. Figure 1 graphically represents these finding for serum ORAC by gender and smoking status.

**Table 1:** Means (± SD), minima and maxima for serum ORAC by gender and smoking status.

			Non Smoker					Smoker				
			
			N	Mean	SD	Min	Max	N	Mean	SD	Min	Max
ORAC	Male	Baseline 1	5	428	115	310	562	3	510	100	439	581
		Baseline 2	5	391	145	239	577	3	157	42	122	204
		Dose 1	5	389	267	116	728	3	757	145	669	924
		Dose 2	5	397	196	167	645	3	643	89	576	744
		Dose 4	5	649	164	510	885	3	309	183	176	518
		Dose 8	5	536	202	243	707	3	626	38	591	667
	
	Female	Baseline 1	8	352	140	111	535	6	534	65	423	587
		Baseline 2	8	311	122	135	447	6	287	91	156	429
		Dose 1	8	407	170	71	623	6	460	231	192	849
		Dose 2	8	372	200	122	774	6	396	186	169	626
		Dose 4	8	466	153	263	672	6	460	104	340	615
		Dose 8	8	382	132	169	577	6	292	169	130	499
	
	All	Baseline 1	13	380	131	111	562	9	527	68	423	587
		Baseline 2	13	337	129	135	577	9	244	99	122	429
		Dose 1	13	401	195	71	728	9	559	246	192	924
		Dose 2	13	380	190	122	774	9	478	197	169	744
		Dose 4	13	527	174	263	885	9	410	144	176	615
		Dose 8	13	438	170	169	707	9	435	216	130	667

Baseline 1 and 2 were taken prior to the administration of study preparation.

Dose 1 = 1 capsule daily for one week; Dose 2 = 2 capsules daily for one week;

Dose 3 = 4 capsules daily for one week; and Dose 4 = 8 capsules daily for one week.

General linear mixed models (SPSS Mixed) with repeated measures were fitted to the data to maximise retention of data on subjects with missed occasions of measurement. Optimal variance-covariance structures (compound symmetry) were fitted to adjust the estimates and their standard errors for the dependency arising from repeated measurements on the same subjects.

### Serum ORAC

The response over doses formed a consistent pattern for female smokers and non-smokers and male non-smokers. Relative to this typical pattern, a number of anomalies were observed in the male smoker data. These data were excluded from further analyses so that models could be focused on estimating the typical response profile.

Two different types of models were fitted to the data. The first treated all effects, group (3 levels - male non-smoker, female non smoker and female smoker) and dose (5 levels) as factors (effects model). The second fitted dose response models that treated group as a factor but dose as a linear (0, 1, 2, 4, 8) component and dose squared (0, 1, 4, 16, 64) as a quadratic component. Dose response models always retained linear and quadratic dose effects even if non significant.

Full models were initially fitted and appropriate non-significant factors were eliminated. All models were fitted as restricted maximum likelihood mixed models with hierarchical decomposition (Type 1) using covariance structures as specified.

The full factorial model is reported in Table 2 and included all main effects (group and dose) and the group by dose interaction, and the main effects only model for serum ORAC together with degrees of freedom and p values. There was a significant dose effect (p = 0.040) for the serum ORAC primary outcome.

**Table 2:** The effects models for serum ORAC with p values (hierarchical method).

		P values	
**Effect**	**df**	**Full Factorial Model**	**Main Effects Model**

Intercept	1	0.000	0.000
Group	2	0.350	0.358
Dose	4	**0.043**	**0.040**
Group * dose	8	0.492	

**Figure 2:**
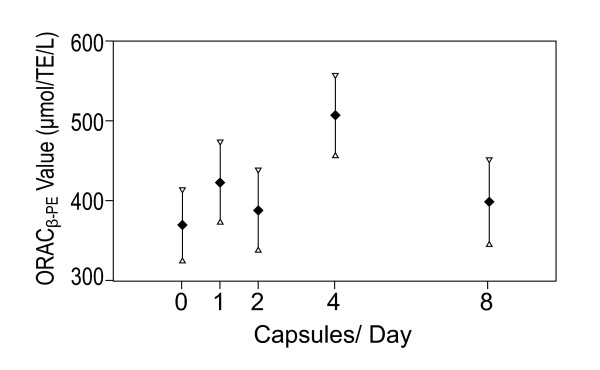
**Serum ORAC predicted means (± SE) by dose(male smokers excluded)**.

Estimated Means and standard errors for dose levels for serum ORAC and p values for Bonferroni adjusted post hoc comparisons with baseline means presented in Table 3. The predicted means (± SE) by dose are presented in Figure 2.

**Table 3:** Serum ORAC estimated means (± SE) and p values for post-hoc comparisons (male smokers excluded).

Dose	Mean	SE	p value for comparison with baseline
Baseline	376.42	31.50	
1	430.15	39.79	0.898
2	395.15	39.79	1.000
4	514.04	39.79	**0.010**
8	405.71	43.09	1.000

There were significant increases in serum ORAC levels from baseline (mean 376.4) in the dose of 4 capsules per day (mean 514.0) p ≤ 0.010, an increase in means of 36.5%. Note that male smokers were not included in these analyses.

### ORAC Dose response models

Progressively fitted models for serum ORAC are reported in Table 4. There was no significant interaction and no significant group effect for the serum ORAC primary outcome. In the final model (Model 3) with any interactions and the group effect eliminated, the quadratic dose effect was significant p ≤ 0.047. The regression parameters results of the final model (Model 3) are reported in Table 5. By differentiating the quadratic equation and equating the derivative to zero, a maximum or minimum dose can be determined. The quadratic equation for serum ORAC is Y = 373.4 + 46.5 * dose - 4.9 * dose * dose. Therefore, *dy*/*dx *= 46.5 - 4.9 * 2 *dose. By equating *dy*/*dx *= *0 *enables one to solve the equation *dose = -46.5*/(-4.9*2). Therefore, the optimal estimated dose is 4.7 capsules per day.

**Table 4:** Progressively fitted models for serum ORAC with p values (hierarchical method) [male smokers excluded].

Effect	df	Model 1	Model 2	Model 3
Intercept	1	0.000	0.000	0.000
Group	1	0.206	0.096	
Linear dose	1	0.158	0.153	0.135
Quadratic dose	1	**0.047**	**0.045**	**0.047**
Linear dose * Group	1	0.210		
Quadratic dose * Group	1	0.959		

**Table 5:** Results for quadratic regression model 3 for serum ORAC.

						95% C I	
						
Parameter	Estimate	SE	df	t	Sig.	Lower Bound	Upper Bound
						316.9	429.9
DOSE	46.54	19.71	97.2	2.36	0.020	7.4	85.7
DOSE_SQ	-4.89	2.43	97.5	-2.01	0.047	-9.7	-0.06

### Immune Function

The secondary outcome measures of immune function were 1) mature t cell numbers; 2) mature b cell numbers; 3) helper t cell numbers; 4) suppressant t cell numbers; 5) natural killer cell numbers; 6) phagocytosis of granulocytes; 7) phagocytosis of monocytes; 8) natural killer cell cytotoxicity; and 9) cox 2 expression. Descriptive statistics including sample size, mean, standard deviation, minima and maxima for these outcome measures are reported in Table 6 by dosage level, gender and smoking status.

**Table 6:** Immune function means (± SD), minima and maxima by dosage level, gender and smoking status.

Secondary Outcome Measures			Non Smoker					Smoker				
			
			N	Mean	SD	Min	Max	N	Mean	SD	Min	Max
Mature t cell Number	Male	Baseline 1	5	1.52	0.31	1.10	1.77	3	2.18	0.62	1.74	2.62
		Baseline 2	5	1.56	0.31	1.19	1.99	3	1.75	0.07	1.68	1.82
		Dose 1	5	1.56	0.29	1.23	1.82	3	1.97	0.28	1.69	2.25
		Dose 2	5	1.50	0.40	1.12	1.99	3	1.81	0.16	1.63	1.94
		Dose 4	5	1.56	0.56	1.11	2.31	3	1.93	0.25	1.64	2.12
		Dose 8	5	1.65	0.54	1.21	2.37	3	2.20	0.39	1.84	2.62
	Female	Baseline 1	8	1.56	0.46	1.00	2.17	6	2.01	0.68	0.97	2.76
		Baseline 2	8	1.59	0.34	1.09	1.99	6	2.01	0.70	0.95	2.98
		Dose 1	8	1.56	0.29	1.07	1.93	6	1.99	0.74	0.80	2.75
		Dose 2	8	1.68	0.39	0.99	2.12	6	1.99	0.67	0.86	2.76
		Dose 4	8	1.55	0.35	0.95	1.97	6	1.99	0.77	0.89	2.97
		Dose 8	8	1.63	0.46	1.02	2.26	6	1.85	0.74	1.11	2.87

Mature b cell number	Male	Baseline 1	5	0.24	0.08	0.17	0.36	3	0.50	0.15	0.39	0.60
		Baseline 2	5	0.22	0.06	0.16	0.31	3	0.37	0.16	0.26	0.55
		Dose 1	5	0.25	0.10	0.14	0.36	3	0.39	0.11	0.32	0.52
		Dose 2	5	0.22	0.06	0.14	0.28	3	0.36	0.14	0.27	0.52
		Dose 4	5	0.22	0.06	0.15	0.30	3	0.36	0.15	0.27	0.53
		Dose 8	5	0.22	0.05	0.19	0.30	3	0.42	0.18	0.29	0.63
	Female	Baseline 1	8	0.24	0.05	0.17	0.34	6	0.39	0.17	0.23	0.59
		Baseline 2	8	0.24	0.05	0.16	0.29	6	0.32	0.16	0.11	0.53
		Dose 1	8	0.25	0.08	0.15	0.37	6	0.32	0.13	0.14	0.50
		Dose 2	8	0.26	0.08	0.16	0.38	6	0.33	0.14	0.17	0.54
		Dose 4	8	0.24	0.06	0.15	0.34	6	0.35	0.20	0.11	0.60
		Dose 8	8	0.25	0.05	0.18	0.30	6	0.34	0.17	0.12	0.49

Helper t cell number	Male	Baseline 1	5	0.99	0.30	0.58	1.23	3	1.27	0.18	1.14	1.40
		Baseline 2	5	0.98	0.31	0.69	1.40	3	1.19	0.31	0.88	1.50
		Dose 1	5	1.01	0.31	0.59	1.28	3	1.32	0.23	1.13	1.58
		Dose 2	5	0.99	0.39	0.63	1.43	3	1.21	0.28	0.99	1.53
		Dose 4	5	1.02	0.47	0.58	1.61	3	1.29	0.37	1.06	1.71
		Dose 8	5	1.09	0.52	0.64	1.76	3	1.53	0.53	1.22	2.14
	Female	Baseline 1	8	0.96	0.29	0.70	1.46	6	1.24	0.39	0.60	1.61
		Baseline 2	8	1.00	0.22	0.73	1.29	6	1.24	0.38	0.61	1.59
		Dose 1	8	0.97	0.15	0.73	1.20	6	1.24	0.46	0.52	1.83
		Dose 2	8	1.06	0.23	0.66	1.33	6	1.23	0.39	0.56	1.75
		Dose 4	8	0.98	0.21	0.62	1.18	6	1.25	0.49	0.59	2.06
		Dose 8	8	1.02	0.27	0.61	1.29	6	1.10	0.29	0.75	1.44

Suppressant t cell number	Male	Baseline 1	5	0.47	0.12	0.28	0.59	3	0.77	0.54	0.39	1.15
		Baseline 2	5	0.54	0.19	0.28	0.81	3	0.54	0.25	0.38	0.83
		Dose 1	5	0.51	0.17	0.27	0.75	3	0.61	0.31	0.42	0.96
		Dose 2	5	0.50	0.17	0.31	0.70	3	0.55	0.23	0.37	0.81
		Dose 4	5	0.52	0.19	0.29	0.72	3	0.59	0.26	0.43	0.89
		Dose 8	5	0.53	0.17	0.31	0.66	3	0.63	0.18	0.49	0.84
	Female	Baseline 1	8	0.55	0.20	0.30	0.89	6	0.77	0.43	0.36	1.50
		Baseline 2	8	0.53	0.16	0.31	0.80	6	0.74	0.45	0.33	1.62
		Dose 1	8	0.54	0.15	0.34	0.79	6	0.72	0.42	0.27	1.50
		Dose 2	8	0.55	0.19	0.30	0.86	6	0.72	0.42	0.29	1.51
		Dose 4	8	0.53	0.16	0.32	0.81	6	0.73	0.41	0.29	1.45
		Dose 8	8	0.56	0.24	0.31	0.98	6	0.77	0.54	0.33	1.55

Natural killer cell number	Male	Baseline 1	5	0.20	0.14	0.08	0.44	3	0.36	0.02	0.34	0.37
		Baseline 2	5	0.22	0.16	0.12	0.50	3	0.27	0.04	0.24	0.31
		Dose 1	5	0.19	0.13	0.11	0.41	3	0.29	0.02	0.28	0.32
		Dose 2	5	0.13	0.04	0.09	0.17	3	0.29	0.03	0.26	0.31
		Dose 4	5	0.16	0.06	0.10	0.21	3	0.30	0.03	0.27	0.32
		Dose 8	5	0.17	0.08	0.07	0.24	3	0.34	0.12	0.26	0.48
	Female	Baseline 1	8	0.18	0.08	0.10	0.32	6	0.21	0.12	0.12	0.41
		Baseline 2	8	0.18	0.06	0.10	0.28	6	0.25	0.15	0.10	0.50
		Dose 1	8	0.19	0.08	0.10	0.35	6	0.21	0.09	0.12	0.37
		Dose 2	8	0.20	0.07	0.11	0.29	6	0.24	0.12	0.11	0.45
		Dose 4	8	0.17	0.06	0.10	0.26	6	0.25	0.09	0.14	0.37
		Dose 8	8	0.18	0.09	0.09	0.31	6	0.32	0.18	0.14	0.56

Phagocytosis of Granulocytes	Male	Baseline 1	5	37.1	6.0	31.0	44.4	3	40.6	1.3	39.6	41.5
		Baseline 2	5	47.6	5.1	39.6	53.2	3	40.4	8.4	31.0	47.2
		Dose 1	5	35.4	3.1	33.0	39.8	3	48.0	4.9	42.4	51.6
		Dose 2	5	48.8	14.3	27.5	58.1	3	49.2	15.0	32.8	62.2
		Dose 4	5	40.4	6.3	30.9	44.5	3	38.6	3.0	35.2	40.6
		Dose 8	5	48.6	3.4	44.5	51.5	3	48.1	5.5	43.7	54.2
	Female	Baseline 1	8	38.8	10.1	27.6	57.3	6	40.0	8.8	27.1	49.3
		Baseline 2	8	51.4	7.4	37.6	60.7	6	53.8	8.0	46.0	65.2
		Dose 1	8	49.3	13.3	33.3	75.7	6	46.3	9.0	32.9	57.9
		Dose 2	8	46.0	13.9	26.6	64.2	6	51.4	13.8	28.6	65.9
		Dose 4	8	39.5	7.3	34.8	51.7	6	48.5	8.8	36.2	58.6
		Dose 8	8	50.9	8.9	39.5	63.7	6	56.3	6.7	47.0	61.9

Phagocytosis of monocytes	Male	Baseline 1	5	31.1	4.5	24.9	35.6	3	29.1	3.5	26.6	31.5
		Baseline 2	5	35.5	2.2	33.3	38.9	3	30.4	9.4	23.9	41.2
		Dose 1	5	27.1	3.2	23.5	32.0	3	35.3	3.1	31.9	38.0
		Dose 2	5	38.4	10.8	22.5	45.7	3	35.4	10.1	25.8	46.0
		Dose 4	5	31.8	5.7	25.1	37.6	3	27.4	1.4	25.8	28.3
		Dose 8	5	37.6	1.8	35.8	39.7	3	32.4	5.4	26.5	37.2
	Female	Baseline 1	8	34.7	8.4	20.0	47.3	6	32.4	5.1	25.3	39.2
		Baseline 2	8	39.5	7.7	28.9	47.7	6	39.3	5.4	30.3	45.1
		Dose 1	8	35.5	8.1	26.0	49.0	6	34.6	6.5	24.8	42.6
		Dose 2	8	33.1	7.3	22.5	45.3	6	33.8	9.5	21.5	49.8
		Dose 4	8	30.9	4.6	25.0	38.6	6	36.2	7.5	30.5	49.8
		Dose 8	8	32.5	5.5	24.0	39.9	6	34.5	8.8	24.4	44.8

Natural killer cell cytotoxicity	Male	Baseline 1	5	15.5	6.0	10.6	25.2	3	22.0	0.9	21.3	22.6
		Baseline 2	5	18.7	5.4	11.0	25.4	3	22.8	17.0	10.7	34.8
		Dose 1	5	18.1	6.8	10.4	24.8	3	22.4	4.5	17.6	26.5
		Dose 2	5	13.7	4.8	7.5	19.3	3	21.8	3.9	17.3	24.7
		Dose 4	5	14.1	4.1	8.9	19.0	3	23.5	6.6	17.6	30.6
		Dose 8	5	15.1	2.8	11.3	17.8	3	23.2	6.0	19.6	30.1
	Female	Baseline 1	8	14.8	5.9	9.1	24.0	6	15.1	4.5	10.6	21.5
		Baseline 2	8	13.9	5.7	8.7	25.6	6	16.5	6.6	8.6	23.4
		Dose 1	8	15.0	5.3	8.9	24.6	6	16.4	4.9	10.4	23.9
		Dose 2	8	14.1	5.2	8.6	21.6	6	16.6	4.5	10.0	20.8
		Dose 4	8	13.4	4.3	7.6	19.1	6	13.3	5.5	4.1	19.3
		Dose 8	8	12.5	5.0	7.8	19.1	6	15.9	7.5	10.5	26.5

Cox 2	Male	Baseline 1	5	78.0	8.9	67.8	89.6	3	83.6	3.9	80.8	86.3
		Baseline 2	5	82.9	2.9	79.7	85.3	3	83.0	2.2	80.6	85.0
		Dose 1	5	82.6	3.1	79.0	86.4	3	85.1	4.1	82.5	89.9
		Dose 2	5	83.2	1.4	82.2	85.3	3	79.6	2.2	78.1	82.1
		Dose 4	5	78.2	5.1	73.8	85.2	3	84.8	5.0	80.9	90.5
		Dose 8	5	79.4	3.6	75.4	83.5	3	85.8	8.5	76.2	92.5
	Female	Baseline 1	8	78.9	5.9	71.8	87.9	6	80.2	5.4	76.3	89.4
		Baseline 2	8	81.0	5.5	72.0	88.5	6	79.2	9.1	61.9	85.8
		Dose 1	8	82.6	3.5	75.9	88.0	6	85.9	3.0	82.7	90.3
		Dose 2	8	83.6	3.8	77.3	89.2	6	81.3	4.0	74.8	86.0
		Dose 4	8	81.0	4.3	73.4	85.2	6	83.7	4.8	78.4	92.3
		Dose 8	8	81.3	5.2	76.1	90.1	6	82.7	5.5	76.9	87.6

Baseline 1 and 2 were taken prior to the administration of study preparation.

General linear mixed models (SPSS Mixed) with repeated measures were fitted to the data to ensure maximise retention of data on subjects with missed occasions of measurement. Optimal variance-covariance structures (compound symmetry or completely general or unstructured) were fitted to adjust the estimates and their standard errors for the dependency arising from repeated measurements on the same subjects.

The main effects factorial model (smoker, gender and dose) for all secondary outcome measures together with degrees of freedom and p values is reported in Table 7. All 2 way and 3 way interactions were non significant. Hierarchical method of fitting was used so that measurements were adjusted for smoking status, and gender before the dose factor was fitted.

**Table 7:** Immune Function p values for the main effects factorial models.

Secondary Outcome Measures	Smoking status	Gender	Dose
Mature t cell number	*0.053*	0.901	0.320
Mature b cell number	**0.029**	0.704	0.839
Helper t cell number *	*0.093*	0.824	0.134
Suppressant t cell number	0.188	0.565	0.806
Natural killer cell number*	**0.004**	0.165	0.179
Phagocytosis of granulocytes*	0.389	**0.036**	**0.000**
(4 capsule dose excluded)	0.607	**0.038**	**0.000**
Phagocytosis of monocytes *	0.872	**0.007**	**0.003**
(4 capsule dose excluded)	0.516	**0.020**	0.094
Natural killer cell activity	0.129	**0.040**	0.345
Cox 2 inhibition *	0.937	0.630	**0.001**

* indicates models were fitted with unstructured variance-covariance otherwise compound symmetry models were fitted.

The fitting of the factorial models resulted in significant smoking status factor for mature b cell numbers (p ≤ 0.028), natural killer cell numbers (p ≤ 0.003) and urinary creatinine (p ≤ 0.029); significant gender factor for phagocytosis of granulocytes (p ≤ 0.035), phagocytosis of monocytes (p ≤ 0.007) and natural killer cell activity (p ≤ 0.040); and significant dose factor for phagocytosis of granulocytes (p ≤ 0.000), phagocytosis of monocytes (p ≤ 0.003) and cox 2 expression (p ≤ 0.001).

The assays for phagocytosis of granulocytes and monocytes were handled by a single operator for all measurements excepting week 4. As these measurements are sensitive to laboratory operator variation it was decided by the study team to undertake separate analysis with the dose of 4 capsules per day excluded. Under these conditions there was a significant dose effect retained for phagocytosis of granulocytes only (p ≤ 0.000).

**Figure 3:**
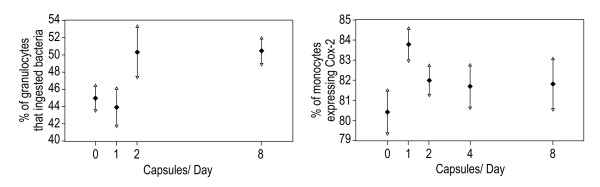
**Immune function variables with a significant dose effect**.

The significant effects together with estimated marginal means, standard errors and significant post hoc comparisons (Bonferroni adjusted) for secondary outcome variables is reported in Table 8. The two variables which had a significant dose effect, phagocytosis of granulocytes (with 4 capsule dose excluded) and cox 2 expression are shown in Figure 3.

**Table 8:** Immune Function significant effects exist and significant post hoc comparisons.

Secondary Outcome Measure	Effect		Mean	SE	Pairwise	p value
Mature B Cell Number	Smoker	No	0.25	0.03		0.028
		Yes	0.35	0.04		

Natural Killer Cell Number	Smoker	No	0.20	0.02		0.003
		Yes	0.27	0.02		

Phagocytosis of Granulocytes	Dose	Baseline	45.01	1.43	base vs 8	0.002
		1	43.70	2.17	1 vs 8	0.038
		2	50.68	2.89	2 vs 4	0.010
		4	42.81	1.65	4 vs 8	0.000
		8	50.49	1.58		
	
	Gender	Male	43.70	2.18		0.038
		Female	49.37	1.73		

Phagocytosis of Granulocytes	Dose	Baseline	45.01	1.44	base vs 8	0.001
(4 capsule dose excluded)		1	43.96	2.19	1 vs 8	0.026
		2	50.36	2.92		
		8	50.47	1.54		
	
	Gender	Male	44.64	2.23		0.035
		Female	50.27	1.78		

Phagocytosis of Monocytes	Dose	Baseline	34.37	0.89	2 vs 4	0.003
		1	33.24	1.38		
		2	35.76	1.83		
		4	32.01	1.27		
		8	32.70	1.41		
	
	Gender	Male	31.43	1.29		0.007
		Female	35.80	1.02		

Phagocytosis of Monocytes	Gender	Male	32.03	1.39		0.022
(4 capsule dose excluded)		Female	35.93	1.09		

Natural Killer Cell Cytotoxicity	Gender	Male	19.41	1.70		0.04
		Female	14.77	1.28		

Cox 2.	Dose	Baseline	80.42	1.06	base vs 1	0.047
		1	83.77	0.79		
		2	81.99	0.73		
		4	81.69	1.07		
		8	81.80	1.26		

### Safety

Participants report of adverse events treated with permitted medication (paracetamol/acetaminophen) included, headache (n = 6), sports related pain (n = 1), and period pain (n = -1). Three participants reported the following adverse events but did not use medication, removal of basal cell carcinoma from the right cheek (n = 1), preparatory injections for overseas travel (hepatitis A and typhoid) (n = 1), flatulence and constipation (n = 1). One subject (35 year old female) treated a cold sore with L-lysine 500 mgs over 4 days during week 4.

Repeated measures analyses using SPSS GLM was used to analyse the baseline and final scores for safety measures for the full factorial model of smoking status, gender and smoking status by gender interaction. Post-hoc comparisons of baseline measurement versus final measurement for any safety variable showing a significant effect was undertaken. There were some significant changes in the safety variables (full blood count, liver function, urea, creatinine and electrolytes) over the course of the trial but these were generally small, well within clinical reference ranges and assessed as not of clinical significance. These were 1) a decrease in platelet count in all subjects (normal adult reference range: 150-400 × 10^9^/L) from an estimated marginal mean of 272.68 to 250.82 (p ≤ 0.005); 2) a decrease in serum potassium in all subjects (normal adult reference range: 3.8-4.9 mmol/L) from 4.46 to 4.26 (p ≤ 0.011); 3) an increase in white cell count in female smokers (normal adult reference range: 4.0-10.0 × 109/L) from 7.40 to 8.42 (p ≤ 0.048); and 4) an increase in alkaline phosphatase (ALP) in male non-smokers (normal adult reference range: 47-136 u/L) from 65.60 to 71.80 (p ≤ 0.050).

A similar analysis was undertaken for vital signs (pulse, systolic and diastolic blood pressure and weight using a main effects factorial model (smoker, gender and dose). Hierarchical method of fitting was used so that measurements were adjusted for smoking status, and gender prior to dose being fitted.

This showed a significant gender factor for systolic blood pressure (p ≤ 0.050) and significant dose factor for both weight (p ≤ 0.003) and systolic blood pressure (p ≤ 0.022). Mean weight reduction of 0.8 kg was observed over the 4-week treatment period. The weight reduction was found not to differ significantly between males and females. Similarly a decrease in mean systolic blood pressure (121 down to 117 mmHg) was also observed. The reduction in systolic blood pressure was also found not to differ significantly between males and females.

## Discussion

A significant increase in antioxidant activity (serum ORAC) was observed between a dose of zero to a dose of 4 capsules per day (p ≤ 0.040). Whilst the observed levels of serum ORAC differed between groups defined in terms of gender and smoking status, the extent of the increase did not differ significantly among these groups (p ≤ 0.492). The percentage increase in the serum ORAC mean from baseline to 4 capsules daily was 36.6%. Given the effect was only seen in both genders in non-smokers further investigation needs to be in this cohort.

A quadratic function for dose levels was fitted in order to estimate a dose response curve for estimating the optimal dose. The quadratic component of the curve was significant (p = 0.047), with predicted serum ORAC scores increasing from the zero dose to a maximum at a predicted dose of 4.7 capsules per day and decreasing for higher doses. This suggests that the optimal dose to assess in a Phase 3 randomized controlled trial is 4 capsules daily.

A few studies have investigated the effects of dietary changes via foods or supplements on serum ORAC. In food studies, increases in serum ORAC have been documented following ingestion of strawberries (14.4% increase) and spinach (28.5% increase)[12], buckwheat honey (7% increase) [13] and concord grape juice (8% increase) [23]. Ingestion of a high-carotenoid content diet had no effect on serum ORAC [24].

The results of dietary supplementation trials on effecting serum antioxidant status have been mixed. In a placebo-controlled trial of healthy adults, a single 100 g dose of wild blueberry powder significantly increased serum ORAC by up to 16% [25] and a single 1.25 g dose of vitamin C raised serum ORAC by 23% [12]. In a second placebo-controlled study of 500 mg/day vitamin C, serum ORAC was significantly increased, but the percent change was not indicated [26]. Additional studies of supplements designed to have antioxidant benefits have demonstrated no effect on serum ORAC: an antioxidant supplement (vitamin E, beta-carotene, ascorbic acid, selenium, alpha-lipoic acid, N-acetyl 1-cysteine, catechin, lutein, and lycopene) [27]; either of two antioxidant supplements (an antioxidant vitamin/mineral table or a vitamin/mineral/fruit and vegetable powder capsule) [24]; or a fruit-based antioxidant drink (MonaVie Active, Salt Lake City, Utah) [28]. The Ambrotose AO capsules in this study which increase serum ORAC by 36% appears to be the most promising antioxidant supplement investigated to date, providing more protection than spinach or high dose vitamin C.

Among the secondary outcome measures, a significant dose effect was observed on phagocytosis of granulocytes, with the increase being significant from a zero dose to dose of 8 capsules per day. Due to laboratory operator variation, the data for dose 4 were deemed to be inconsistent with the remaining data and excluded from analysis. It was not possible therefore to determine whether there was a significant increase at a dose of less than 8 capsules per day. This represents a 12% increase from baseline and suggests that the preparation may have an immunomodulatory effect by improving the non-specific, anti-infective mechanisms of defence. The study was not powered for measuring the secondary outcomes which were exploratory in nature and the fact that there was no change in the lymphocyte subset counts or in the other two markers of non-specific immune response, phagocytosis by monocytes or in natural killer cell cytotoxicity may be a type II error and these results, therefore, cannot be considered conclusive.

A significant increase was also observed on Cox 2 expression between a zero dose and a dose of 1 capsule per day, with Cox 2 expression decreasing from this high point at higher doses but remaining above the zero dose level though none of the other comparisons were significant. The inducible form of cyclooxygenase, COX-2, is an immediate-early response gene with complex regulation that plays an essential role in vascular homeostasis and inflammation. Pharmacological manipulation of COX-2 activity can have both beneficial and problematic clinical effects depending on the substrates available in the cell membrane [29], though the increase of 4% seen in this study is unlikely to be of any clinical significance.

The preparation was demonstrated to be safe over the course of the study. Adverse events experienced during were mild and self limiting, there were no changes in the haemopoietic, liver or renal systems of clinical significance and the vital signs remained healthy. Unanticipated changes in systolic blood pressure and weight moved in a generally beneficial direction but were not of any real clinical significance.

The most significant limitation of this study was that it was open label combined Phase 1 and 2 trial and is subject to potential bias that is reduced with the use of randomization and blinding.

## Conclusion

It is important that this study is not over interpreted; it aimed to find an optimal dose to assess the study medication using a more rigorous clinical trial design. The study achieved this aim and demonstrated that the study medication has the potential to increase antioxidant activity in non-smokers. These activities now need to be demonstrated in a Phase 3 randomised double-blind placebo-controlled trial in non-smokers (RCT).

## Competing interests

The study was sponsored by Mannatech Inc (USA) under contract to Southern Cross University and performed independently by NatMed-Research. Mannatech Inc (USA) paid the article-processing charge associated with the publication of this paper.

## Authors' contributions

SPM was the principal investigator and was involved in the study design, management of the clinical trial, interpretation of the results and preparation of the manuscript; LS supervised the laboratory measurements, was involved in study design and interpretation of results and contributed to the manuscript; PAC was involved in study design and interpretation of results and contributed to the manuscript; JO was the study co-ordinator and was involved in the study design, the day to day management of the clinical trial, data entry and contributed to the manuscript; LB supervised the statistical analysis and was involved in the interpretation of the results and contributed to the manuscript; MR undertook the statistical analysis and was involved in the interpretation of the results and contributed to the manuscript; PC undertook the immune function studies and was involved in interpretation of the results and contributed to the manuscript; and CM directs the laboratory where the immune studies and serum ORAC was undertaken and contributed to the manuscript. All authors read and approved the final manuscript.

## Pre-publication history

The pre-publication history for this paper can be accessed here:

http://www.biomedcentral.com/1472-6882/10/16/prepub
